# PeptoGrid—Rescoring Function for AutoDock Vina to Identify New Bioactive Molecules from Short Peptide Libraries

**DOI:** 10.3390/molecules24020277

**Published:** 2019-01-13

**Authors:** Arthur O. Zalevsky, Alexander S. Zlobin, Vasilina R. Gedzun, Roman V. Reshetnikov, Maxim L. Lovat, Anton V. Malyshev, Igor I. Doronin, Gennady A. Babkin, Andrey V. Golovin

**Affiliations:** 1 Faculty of Bioengineering, Lomonosov Moscow State University, Moscow 119234, Russia; alexander.zlobin@fbb.msu.ru (A.S.Z.); golovin.andrey@gmail.com (A.V.G.); 2 Ineractomics Lab, Institute of Molecular Medicine, Sechenov First Moscow State Medical University, Moscow 119146, Russia; r.reshetnikov@gmail.com; 3 Shemyakin and Ovchinnikov Institute of Bioorganic Chemistry of the Russian Academy of Sciences, Moscow 117997, Russia; 4 Lactocore Inc., Plymouth, MI 48170, USA; vrgedzun@gmail.com (V.R.G.); malyshev@lactocore.com (A.V.M.); doronin@lactocore.com (I.I.D.); gbabkin3@mail.ru (G.A.B.); 5 Department of Human and Animal Physiology, Faculty of Biology, Lomonosov Moscow State University, Moscow 119899, Russia; lovat@mail.ru; 6 Faculty of Computer Science, National Research University Higher School of Economics, Moscow 101000, Russia

**Keywords:** docking, peptides, rescoring, gabab receptor, *Danio rerio*

## Abstract

Peptides are promising drug candidates due to high specificity and standout safety. Identification of bioactive peptides de novo using molecular docking is a widely used approach. However, current scoring functions are poorly optimized for peptide ligands. In this work, we present a novel algorithm PeptoGrid that rescores poses predicted by AutoDock Vina according to frequency information of ligand atoms with particular properties appearing at different positions in the target protein’s ligand binding site. We explored the relevance of PeptoGrid ranking with a virtual screening of peptide libraries using angiotensin-converting enzyme and GABAB receptor as targets. A reasonable agreement between the computational and experimental data suggests that PeptoGrid is suitable for discovering functional leads.

## 1. Introduction

Peptides are a class of molecules that serve as hormones, neurotransmitters, growth factors, ion channel ligands, and anti-infective agents [1]. Peptides are selective and efficacious signal molecules with a standout safety and tolerability profiles, which makes them promising drug candidates. Novel synthetic strategies allow for cost-effective production of peptide drugs and optimization of pharmacodynamic and therapeutic properties of peptide leads through chemical modifications of component amino acids [2].

There are several approaches to peptidic drug development, from the isolation of natural peptides from biological tissues of different origin to rational design strategies based on the known crystal structures. As an alternative to the knowledge-based approaches, many methods to identify bioactive peptides de novo have been developed, such as phage display [3] and combinatorial library techniques [4]. Computational methods represent a cost-effective tool for high-throughput screening of peptide libraries [5], among which the docking-based virtual screening approach is the most widely used [6]. Among the main challenges in a molecular docking are the drawbacks of current scoring methods [7]. For computational simplicity, scoring functions usually estimate rather than calculate binding affinities, which leads to the inaccurate ranking of docking results [8]. To facilitate screening of large molecular libraries, a tiered scoring scheme is often employed whereby a simple scoring function is used as a fast filter of the library, and a more rigorous and computationally expensive scoring function is used to rescore the top hits to produce the final list of ranked models [9,10].

Here we present the PeptoGrid algorithm, a novel rescoring procedure for AutoDock Vina [11] predictions of peptide-protein complexes. It is based on the frequency information of ligand atoms with particular properties (aromatic carbon, hydrogen bond donor/acceptor et cetera) appearing at different sites of a docking box.

PeptoGrid procedure has a number of features that should be considered while choosing the rescoring algorithm for a system under study: (i) it works optimally for a large peptide library, which allows for a more detailed characterization of a protein’s binding site; (ii) rescoring is possible only within a closed set of peptides; (iii) current implementation is limited to peptide ligands consisting of non-modified amino acids.

To prove the feasibility of the rescoring function, we used the angiotensin-converting enzyme (ACE) as a benchmark system for comparison of the predicted ranking of peptidic ACE inhibitors with their experimentally determined inhibitory concentrations. We also performed a virtual screening of biogenic peptides capable of binding the extracellular domain of the GABAB receptor, a drug target for treatment of addictions, depression and anxiety disorders [12]. We validated the results of computational prediction with in vivo experiments on zebrafish (*Danio rerio*) test systems. The best-scored compounds had a pronounced effect on the zebrafish behavior.

## 2. Results and Discussions

### 2.1. PeptoGrid Algorithm

Peptide docking is a non-trivial task that has no generally accepted solution. A large number of internal degrees of freedom and a vast chemical diversity due to the size of amino acid alphabet distinguish peptides from small molecules [13]. Current evaluation functions are poorly optimized for peptide ligands, which often results in the discrepancy between the poses with the best geometry and their scores [14]. The problem becomes even more pronounced when comparing peptides from large-scale libraries [15]. To address that, alternative scoring functions were designed that could rearrange the obtained ligand poses, for example, based on molecular mechanics energies combined with the Poisson–Boltzmann (MM/PBSA) [9] or generalized Born and surface area continuum solvation (MM/GBSA) [16] with molecular dynamics simulations, using consensus (combining several scoring functions) [17] or machine learning approaches [18].

Here we present PeptoGrid, a novel rescoring approach for peptide ligand poses obtained with AutoDock Vina program [11]. AutoDock Vina allows for a thorough scanning of ligand’s conformational space, providing a high probability of obtaining optimal (close to observed in crystal structures) geometries of complexes with a target protein [19]. AutoDock Vina is well suited for peptide docking, producing reasonable predictions for up to four amino acids-long ligands [13].

The aim of the PeptoGrid rescoring is to rearrange the results of Autodock Vina predictions for the peptide-protein complexes according to the frequency of appearance of ligand atoms with specific physicochemical characteristics at a given position in a protein’s ligand-binding site.

The starting point of the PeptoGrid rescoring algorithm is an assignment of an atom type to the ligand’s atoms according to the Tripos SyBYL MOL2 format (Figures S1 and S2). A 3D grid with a step of 0.1 A is built for a docking box. For every grid cell, the frequency of occurrence of each atom type is estimated based on coordinates from all docking poses. The size of an atom is determined by its van der Waals radius, therefore a single atom fills several grid cells. A weighting scheme is obtained from the spatial distribution of atom types inside the ligand-binding site of the protein. After that, the weighting scheme is applied to the individual ligand poses. A score for a ligand atom is assigned according to the value in the nearest grid vertex, and a pose score Sr is a sum of all its atoms’ scores normalized by the number of atoms in the peptide:

(1) $$Sr=\frac{\sum_{i}^{N}sa_{i}}{N}$$

where *N* is a total number of atoms in the peptide, $sa_{i}$ is a value of the scoring function for an individual atom *i*. For a final ranking of the poses our algorithm performs a normalization to the maximum Sr value to scale total score range from 0 to 1.

The direct consequence of using the SyBYL MOL2 format while building atom type frequency maps is that PeptoGrid algorithm could be adapted for ligand libraries composed of other organic compounds, for example, nucleic acids. It makes the proposed algorithm compatible with in silico SELEX. We plan to implement this approach in the near future.

### 2.2. Benchmark System

Since our rescoring function was tailored for peptide ligands, we used AHTPDB (antihypertensive inhibiting peptide database) as a source of experimental data for validation of the PeptoGrid algorithm. AHTPDB contains about 1700 individual peptides, almost all of which target angiotensin-converting enzyme (ACE) [20]. We used the structure of ACE in complex with phosphinic tripeptide (PDB ID 2XY9 [21]) as a source of receptor coordinates. Since the structure contained two tripeptides in the active site, we converted our dataset into tripeptide library (see Materials and Methods). The total size of the dataset combined from AHTPDB and the library was 432 unique tripeptides (Figure S3).

The PeptoGrid algorithm was initially designed to rescore AutoDock Vina predictions; to test the PeptoGrid performance we also used alternative docking packages in this study, namely PLANTS and LeDock. PLANTS is a versatile docking package which implements efficient sampling algorithms as well as several scoring functions [22]. LeDock was recently claimed to be the most efficient free docking tool, outperforming AutoDock Vina and most of the commercial packages [19]. We also investigated whether PeptoGrid was able to improve the predictions of PLANTS and LeDock, for which we modified the code to make it compatible the packages.

Docking simulations in PLANTS and LeDock yielded 8640 individual tripeptide poses each, while due to intrinsic restrictions there were 8602 poses predicted by AutoDock Vina. Pose scores had a normal distribution for all the docking packages (Figure S4A–C). Contrary to that, distribution of the PeptoGrid normalized scores for AutoDock Vina predictions had two bell-shaped peaks with the median values of 0.15 and 0.6 correspondingly (Figure S4D). Note that the source of receptor coordinates, PDB entry 2XY9, represents a previously unknown dual binding of tripeptide inhibitors to ACE primary and secondary binding sites [21]. In all preceding ACE structures, both free and bound to inhibitors, the secondary site was occupied by a string of bound water molecules [21,23]. The first peak on the histogram mainly corresponds to the poses spread in the secondary site, while the second peak almost exclusively consists of scores for the primary site binding modes (Figure S4D). Thus, PeptoGrid rescoring allowed to detail the screening results by separating the two subsets of ligand poses bound to alternative binding sites.

**Figure 1:**
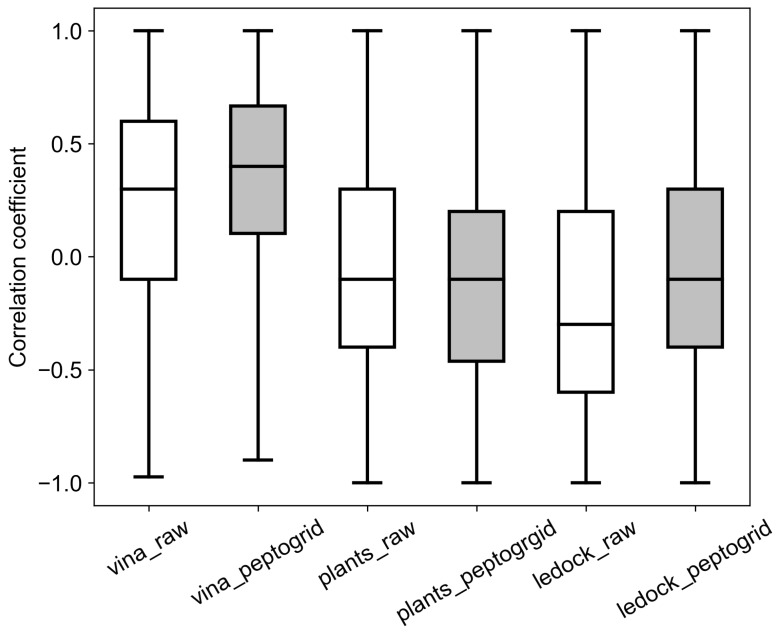
Boostrap analysis of Spearman’s correlation coefficient. PeptoGrid values are in gray.

In order to compare scoring rankings with experimental IC_50_ values from AHTPDB, we performed a statistical analysis using the bootstrap method. The range of IC_50_ values (1–1000 μM) was divided into five intervals of 200 μM each. Bootstrap samples consisted of five IC_50_ values randomly selected from each interval. Spearman’s rank correlation coefficient was then calculated between the poses score ranking and sorted IC_50_ concentrations for the corresponding tripeptides. We performed 1000 such bootstrap samples. AutoDock Vina had a Spearman’s rank correlation coefficient with experimental data of 0.3, while PLANTS_CHEMPLP_ and LeDock showed a weak negative correlation with the medians at −0.1 and −0.3, correspondingly (Figure 1). PeptoGrid rescoring provided better positive correlation with the experimental data for AutoDock Vina and LeDock predictions, shifting the median values to 0.4 and −0.1, correspondingly, while there was no observable improvement for PLANTS_CHEMPLP_ predictions (Figure 1).

**Figure 2:**
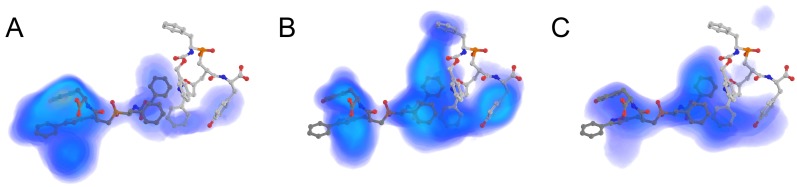
Occurence maps for the C.ar atom type (e.g., aromatic carbon) inside angiotensin-converting enzyme binding site for (**A**) AutoDock Vina, (**B**) PLANTS and (**C**) LeDock. Density increases from blue through yellow to purple. Phosphonic tripeptide from 2XY9 is shown in sticks model.

To address observed differences between the packages and weak correlation with the experimental data we compared PeptoGrid atomic maps based on the geometries of the docking poses calculated for all three packages. In the case of AutoDock Vina there was a good agreement between the conformations of phosphinic tripeptide inhibitors observed in the ACE primary binding site and several atom types. The best correspondence was observed for aromatic atoms (Figure 2A), aliphatic carbons and carbonyl oxygens (not shown). Interestingly, there is a notable difference even for aromatic carbons, which is the direct consequence of the difference in sampling algorithms implemented in AutoDock Vina, PLANTS and LeDock (Figure 2A–C). Unlike AutoDock Vina the other two packages did not give a preference to docking poses located in the primary binding site (Figure 2B,C), which seems to have a more significant effect on the enzyme activity. Thus, despite the very high-quality coverage of the ACE binding site in this area, both packages had problems with detection of functional ligands (see Figure 1). It also explains why PeptoGrid rescoring failed to improve the correlation between PLANTS_CHEMPLP_ ranking and experimental data; there was not enough data on the ligand atoms appearance in the primary site.

Despite the weak correlation with the experimental data, top 10 predictions of all docking packages contained several peptides with known IC_50_ values (Table 1). Application of PeptoGrid rescoring increased the number of such peptides; in case of AutoDock Vina, it resulted in the identification of a CGY peptide with the lowest IC_50_ value in the dataset— 1.3 μM. For the all three packages PeptoGrid rescoring led to a complete change of the top scored tripeptides, which increased the similarity between PLANTS and LeDock predictions from one to three common peptides (Table 1). Between them, GTG tripeptide was the most promising ligand with IC_50_ value of 5.5 μM. The similarity with the AutoDock Vina rescoring was less pronounced due to the different coverage of the binding site (see Figure 2) sharing the single GHG peptide with PLANTS top hits after rescoring (Table 1).

PeptoGrid atomic maps allow to decipher the top hits lists. For example, distinct compact aromaticity zone in AutoDock Vina case can explain the prevalence of GxW peptides over GGY (the absolute best from the dataset). Atomic maps also make it possible to directly visualize the binding site regions with the highest affinity for particular atom type. Once obtained, such maps can be used for rescoring of complexes with even longer peptides, for example, penta- or hexapeptides, though such complexes have to be obtained with different approaches, for instance, incremental docking [24]. Thus, potential applications of PeptoGrid algorithm are not limited to rescoring of molecular docking predictions; it could also be used for rational lead optimization.

### 2.3. Screening System

Actual virtual screening run was performed using a tailored tetrapeptide library (Figure S5) and the GABAB receptor as a target. According to clinical trials results, GABAB agonists confidently reduce anxiety symptoms [25]. The mechanisms associated with the activation of GABAB receptors are still not fully understood because of the widespread distribution of GABAB receptors in various central nervous system compartments [26] and complexity of interactions of GABAB signaling with other types of mediator systems [25].

As a target for molecular docking, we used a structure of GABAB receptor apo-form, PDB ID 4MQE [27]. GABAB functions as a heterodimer assembly of two subunits GBR1 and GBR2. GABAB agonists and antagonists interact with the interdomain crevice of GBR1, which we used as a search space for docking of tetrapeptides [27].

**Figure 3:**
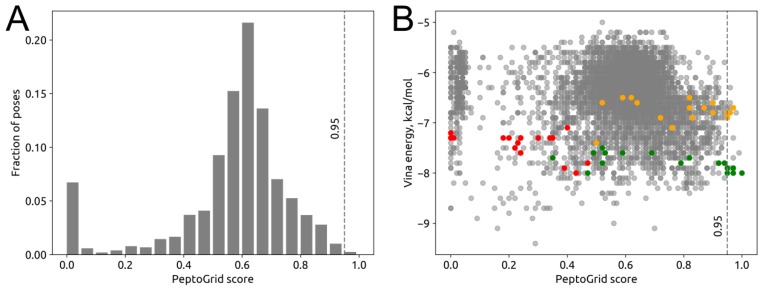
Distribution of PeptoGrid scoring values for GABAB receptor. (**A**) Histogram of PeptoGrid scores. (**B**) Comparison of PeptoGrid score with AutoDock Vina energies. Colors denotes peptides: green is for PYYA, yellow for PSYG and red is for LNPW.

Docking simulation for the tetrapeptide library yielded 5410 individual ligand poses. The geometry of the poses was used to build occurrence frequency maps for each atom type within the binding site (Figure 2B). The maps then were used for rescoring and obtaining of a ranked list of the poses. Distribution of the PeptoGrid normalized scores had a bell-shaped form with a center at 0.65 (Figure 3A). For further analysis, we selected the poses that fall into the area after 95th percentile (Figure 3A). The list contained 20 unique poses (Table S1). Between the top hits, two peptides with sequences PSYG and PYYA had the most poses, 6 and 5 correspondingly. In a further analysis, we used a pose with a lowest PeptoGrid score as a negative control.

The structures of complexes between GABAB and the top hits shared a number of common features. The best-scored poses contained aromatic amino acids, namely tyrosine and phenylalanine. These amino acids were located in the two pockets formed by GABAB residues responsible for signaling function and interactions with agonists and antagonists [27].

We compared the results of PeptoGrid rescoring with the initial ranking of the poses performed by AutoDock Vina program (Figure 3B). There was a low negative correlation between the rankings (Spearman correlation coefficient −0.18). Note that according to the AutoDock Vina binding energy estimates, the negative control peptide LNPW had an affinity for GABAB that was comparable with PYYA and both peptides were more specific against the target protein than PSYG (−8.0, −8.0 and −7.4 kcal/mol, correspondingly).

For further in vivo experimental validation, we selected two PeptoGrid top-scoring peptides with sequences PSYG and PYYA. To test the ability of our algorithm to distinguish binders from inactive compounds, we also added to the set the LNPW peptide with the lowest PeptoGrid score.

### 2.4. Experimental Validation of the Virtual Screening Results

Adult zebrafish (*Danio rerio*) display a repertoire of behaviors which have been characterized both physiologically and pharmacologically [28]. Drug-sensitive zebrafish phenotypes of anxiety have been described and pharmacologically and behaviorally validated [29,30,31]. The GABAergic system of zebrafish was also shown to be similar to human and responsive to known compounds [32,33]. In our experimental studies we used the following assays:

1. Open field test (OFT). Inactivity or swimming on the periphery of the fish tank is connected with the anxiety-like state. Alternatively, exploring the center of the arena is associated with boldness.
2. Shoal cohesion test (SCT). Zebrafish prefer swimming in pods, and this behavioral strategy is thought to be effective against predators. Avoidance of group aggregation (shoal cohesion) is associated with an anxiolytic response.

**Figure 4:**
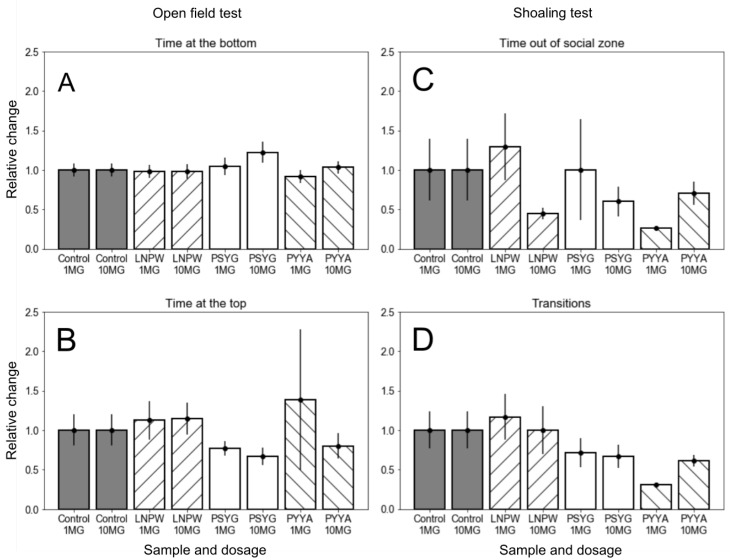
Behavioral effect of the tetrapeptides. Open field test: (**A**) Time at the bottom and (**B**) Time at the top. Shoaling test: (**C**) Time out of social zone and (**D**) Number of transitions. All values are normed to control. 1 MG and 10 MG denote doses of 1 mg/kg and 10 mg/kg correspondingly.

In OFT, both leader peptides PSYG and PYYA produced a minor dose-dependent anxiogenic effect (Figure 4A,B). Introduction of the peptides resulted in a slight increase of the time spent at the bottom of the arena (Figure 4A) and a corresponding decrease of the time spent at the surface (Figure 4B). Note that low dose (1 mg/kg) of PYYA peptide produced a high variety of behavioral effects, which was expressed in a significant diversity of the time spent at the top of the tank (Figure 4B), whereas high dose (10 mg/kg) of the peptide had no such effect. The LNPW peptide produced no dose-dependent effects in OFT. Neither of the peptides in any concentration affected the motor activity of the fish.

In SCT, all ligands, including the negative control peptide LNPW, produced a dose-dependent effect on the time spent by the fish out of the social zone (Figure 4C). Peptides LNPW and PSYG in high doses produced a statistically significant anxiogenic effect, which inversely was observed for the low dose and disappeared in the high dose of PYYA (Figure 4C). The other behavioral component observed in this test, the number of transitions between the three conditional zones, was not affected by LNPW and PSYG peptides, while PYYA in low dose caused a statistically significant decrease of this indicator (Figure 4D). Since the peptides did not inhibit the motor activity of the fish in previous experiments, we associate this behavior with an anxiolytic effect of the peptide, which decreased with an increase of the dose (see Figure 4D).

Generalization of in vivo results should be carried out with caution, especially in the case of the second assay, since the effects of the same peptide on different behavioral components could be controversial (see Figure 4). Maximino and colleagues showed that some behavioral components are more strongly affected by drug treatments [34]. It might explain the discrepancy between the effects of peptides on time spent out of the social zone and the number of transitions between the zones. We could not judge which of the components reflect the real effect of the peptides, while it should be noted that LNPW peptide did not produce a statistically significant effect on any of the behavioral components except the time spent out of the social zone. By contrast, peptides PYYA and PSYG produced anxiogenic effects in most assays.

## 3. Materials and Methods

### 3.1. Preparation of Peptide Libraries

Experimental data for peptides with experimentally measured ACE inhibition efficacy was obtained from AHTPDB database [20] and further filtered to exclude peptides without exact IC_50_ values as well as peptides with multiple diverse values. Upper IC_50_ threshold was set to 1000 μM to exclude outliers with possible erroneous values with overestimated constants. Lower threshold was set to 1 μM. Clean tripeptide library contained 166 peptides.

Library of peptides obtained from hydrolysis of *Bos Taurus* milk was provided by Lactocore Inc., Plymouth, MI, USA. Major fractions were identified and converted into 274 unique tetrapeptides and 289 tripeptides using a sliding window with a step of one amino acid. The intersection between tripeptides from AHTPDB and milk hydrolysate was 23 peptides, thus the total set of unique tripeptides was 432 of 8000 combinatorially possible variants. Logo pictures of datasets were generated with WebLogo version 3.5.0 [35].

### 3.2. Peptide Structures

Initial 3D structures of peptides in PDB format were created with PeptideBuilder [36] and further protonated and undergone energy minimization in a vacuum in GROMACS [37] molecular modeling package with amber99sb-ildn force field [38]. Minimization was performed with Steepest Descent with a threshold of 500 kJ·mol^−1^·nm^−1^.

### 3.3. Docking with AutoDock Vina

Structures of peptides and target were prepared (including removing of water and other heteroatoms and assignment of partial charges) for docking with prepare_ligand4.py and prepare_receptor4.py utilities from AutoDock Tools [39] with the default settings. For ACE we placed docking box to cover both tripeptides in the initial structure (PDB ID 2XY9). The total size of the cubic docking box was set to 30 angstroms along each direction. For GABAB we designated the docking box to cover both GABAB GBR1 and GBR2 subunits with its center placed into the interdomain crevice of subunit GBR1 where agonists and antagonists bind. Box measurements were set to be 35 angstroms along each dimension. Molecular docking simulations were performed with AutoDock Vina [11,40]. “Exhaustiveness” parameter corresponding to the amount of sampling effort was set to 256, and the maximum amount of poses to report was set to 20.

### 3.4. Docking with LeDock

Receptor structure was prepared with the recommended LePro [19] utility and protonation was corrected to correspond to the structure obtained for AutoDock Vina. Docking box was set to the same coordinates, the number of output structures to 20, internal clustering criteria to the recommended value of 1 angstrom. Structures of the ligands were converted into the required mol2 format with OpenBabel version 2.3.2 [41]. LeDock version 1.0, Lephar Research, Stockholm, Sweden was used [19].

### 3.5. Docking with PLANTS

Receptor structure in the required mol2 format was converted from the structure, prepared for the LeDock with the recommended SPORES [42] utility. The docking sphere was set to inscribe docking box used for AutoDock Vina, with the same center and radii of 12.990 angstroms. All settings were set to the recommended defaults: CHEMPLP scoring function was selected, sampling_speed was set to speed1 value, the number of poses to 20, internal clustering criteria was set to the recommended value of 2 angstroms. Structures of peptides in the required mol2 format were the same as for the LeDock. PLANTS version 1.2 64 bit version was used [22].

### 3.6. Rescoring

For subsequent analysis, all poses regardless of their sequence were treated altogether. 3D spatial grid with 0.1 Å step size was built inside the docking box. Each atom was assigned its atom type according to Tripos SYBYL mol2 format as implemented in ODDT [43] package with OpenBabel [41] backend. For every grid cell, the frequency of occurrence of each atom type was estimated based on coordinates from all docking poses with regard to its van der Waals radius [44]. The pose was scored as a sum of all its atoms’ scores normalized by their amount, with single atom’s score being assigned according to the nearest value in the grid. Spatial maps were stored and processed with HDF5 library and H5PY [45] Python interface to this library. Matrix operations were carried out with NumPy [46]. Spatial frequency maps and biomolecular structures were visualized with PyMol [47]. For PLANTS and LeDock the procedure was modified only in the pose reading part.

### 3.7. Animals and Housing

Zebrafish were kept in the ZebTEC (Tecniplast S.p.a, Buguggiate, Italy) recirculating system at 28 ${}^{\circ }C$, pH 6.8–7.5 and osmoticity 550–700 mOsm/L on a 14/10-h light/dark cycle and constant aeration. Feeding was carried out twice a day with a special feed for fish. During the experiment, feeding was carried out on the evening before and in the evening after it. The study was conducted in accordance with EU Directive 2010/63/EU.

### 3.8. Preparation and Admission of Compounds

Peptides PYYA (Pro-Tyr-Tyr-Ala), PSYG (Pro-Ser-Tyr-Gly), LNPW (Leu-Asn-TyrPro-Trp) were provided by Lactacore Inc., Dover, DE, USA. For the in vivo testing in a Zebrafish behavioral test system, doses of 1 mg/kg and 10 mg/kg were used. The dissolution of the peptide from the dry sample was carried out in 0.9% NaCl solution. Anesthesia and immobilization of animals were carried out by placing them in water at a temperature of 10 ${}^{\circ }C$. Injections were carried out intraperitoneally with an insulin syringe 10 min before testing. The fish in the control group were injected with an equivalent volume of solvent.

### 3.9. Behavioral Assays

Open field test (OFT). 20–30 s before the fish were placed in the test tank (Figure S6), video recording was turned on (background shooting). The test fish was placed in an open-field aquarium with a net. The recording was carried out for five minutes.

Shoal cohesion test (SCT). Simultaneously with the placement of the fish in the apparatus (Figure S7), the camera was turned on, and recording was carried out for 5 min. During the processing of records, the residence time and the number of entries in three conditional zones of the aquarium of equal size—about the “flock”, in the middle of the aquarium, and near the opposite wall—were recorded.

### 3.10. Data Analysis

Video recordings for OFT were processed using Noldus Ethovision XT software (version 8.5) (Noldus, Leesburg, VA, USA). The program measures the distance traveled by the animal, its speed, the number of entries into the three conditional zones of the aquarium: “bottom”,“center” and “middle” (respectively, lower, middle and upper third of the aquarium), the time spent in these zones, and latent period of entry into the middle and to the surface of the aquarium. Video recordings for SCT were processed using the RealTimer software (RPC OpenScience Ltd., Moscow, Russia). For SCT, the time spent near the shoaling compartment, the time outside this compartment, as well as the latent period of approaching and moving away from the “flock” were evaluated.

Statistical data processing was carried out using Graph Pad Prism 6 software (Graphpad Software Inc., La Jolla, CA, USA) and Matplotlib library [48]. Data is visualized in the form of mean, the spread is reported in the form of the standard error of the mean.

The normality of the distribution of the obtained data was evaluated. For comparison of variables which were not distributed normally, a non-parametric Mann-Whitney test was applied. Since a paired control measurement was recorded for each compound, the use of this test was valid.

## 4. Conclusions

In summary, we have developed a novel rescoring algorithm PeptoGrid for AutoDock predictions of protein-peptide models. PeptoGrid performs rescoring according to the frequency of appearance of ligand atoms with specific characteristics at a given position of a docking box. This approach allows for a detailed characterization of both the physicochemical properties and the geometry of the target protein’s binding site. PeptoGrid rescoring of ACE peptidic inhibitors’ docking poses allowed to improve and detail AutoDock Vina predictions. The relevance of PeptoGrid ranking of predicted ligand poses was also in a reasonable agreement with in vivo testing on *Danio rerio* model system. The PeptoGrid algorithm allows to discover functional leads reliably and could potentially be adapted for nucleic acids and other organic ligands.

## Figures and Tables

**Table 1:** Unique peptides with experimental IC_50_ values from top 10 predictions

Sequence	IC_50_, μM	Sequence	IC_50_, μM
AutoDock Vina		PeptoGrid	
AWW	6.5	GAW	240.0
VWY	9.4	GHG	122.0
		FGG	82.5
		GGY ^*^	1.3
		GTW	464.5
		GVW	240.0
PLANTS		PeptoGrid	
KFY	45.0	AGS	527.9
YKW	13.3	VAA	13.0
RFH	330.5	GAP	9.3
IWH	3.5	GTG	5.5
		GHG	122.0
		AVV	66.6
LeDock		PeptoGrid	
WYS	500.0	AGS	527.9
KFY	45.0	GAP	9.3
FWN	18.3	GPA	405.0
FNQ	333.5	GTG	5.5
		GPV	4.7

^*^ The best peptide from the dataset.

## Supplementary Materials

The following are available online, Figure S1: Scheme of the docking preparation workflow, Figure S2: Scheme of the PeptoGrid grid calculation and scoring workflow.Scheme of the PeptoGrid grid calculation and scoring workflow, Figure S3: Logo of the tripeptide dataset used for ACE benchmark system, Figure S4: Distribution of docking scores, Figure S5: Logo of the tetrapeptide dataset used for GABAB screening system, Figure S6: Scheme of the open field test tank, Figure S7: Scheme of the shoaling test tank, Table S1: Number of occurrences of peptides from top 20 poses. The PeptoGrid software is available at https://github.com/aozalevsky/peptogrid.
