# Supplementary material for: PeptoGrid—Rescoring Function for AutoDock Vina to Identify New Bioactive Molecules from Short Peptide Libraries
Source: Molecules. 2019 Jan 13;24(2):277. doi: 10.3390/molecules24020277 (PMC6359344; doi:10.3390/molecules24020277)
Supplement: Supplementary file 1 [file molecules-24-00277-s001.pdf]

Supplementary Materials: PeptoGrid — rescoring function for AutoDock Vina to identify new bioactive molecules from short peptide libraries

Arthur O. Zalevsky[1,2,3]*, Alexander S. Zlobin[1,3], Vasilina R. Gedzun[4], Roman V. Reshetnikov[2], Maxim L. Lovat[5], Anton V. Malyshev[4], Igor I. Doronin[4], Gennady A. Babkin[4] and Andrey V. Golovin[1,2,6]

1 ## 1. Computational workflow

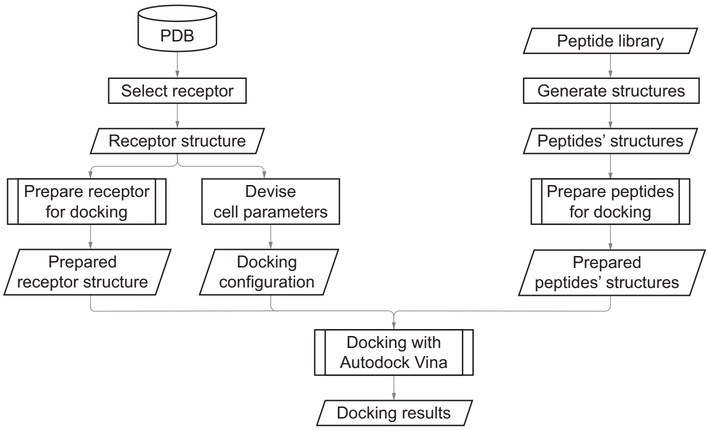

Figure S1. Scheme of the docking preparation workflow.

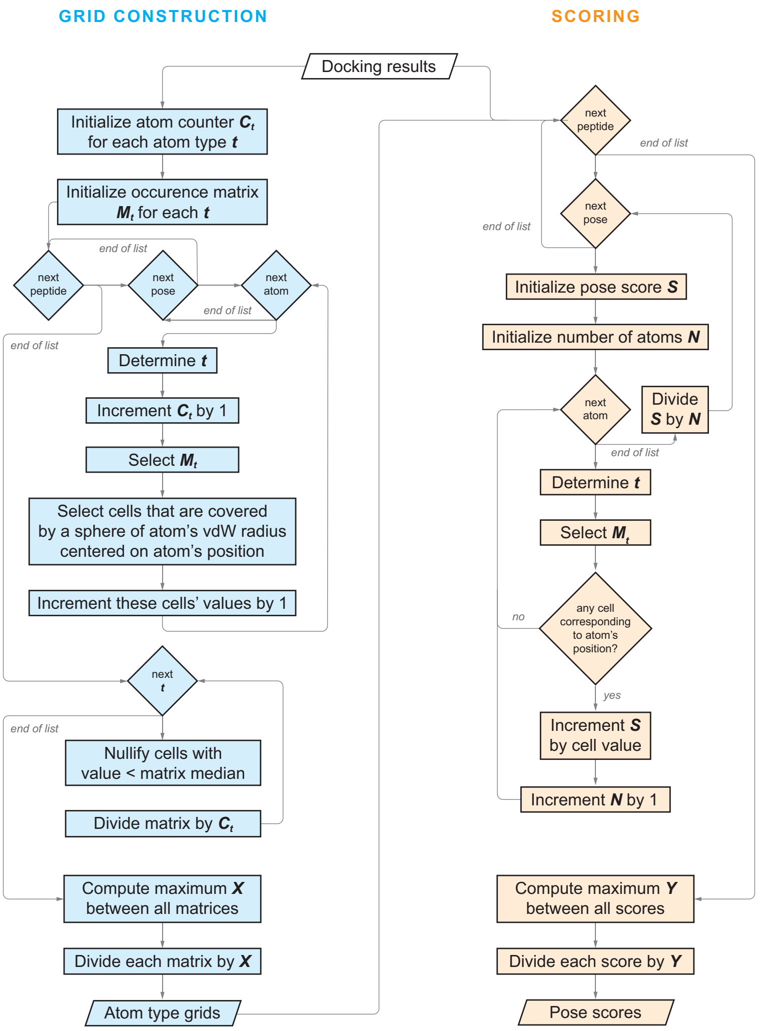

Figure S2. Scheme of the PeptoGrid grid calculation and scoring workflow.

2. Peptides

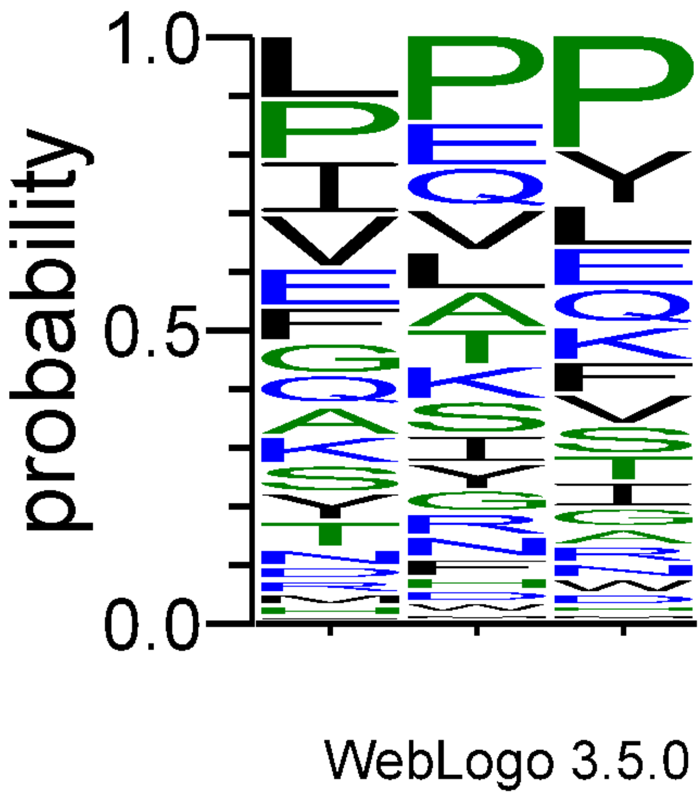

Figure S3. Logo of the tripeptide dataset used for ACE benchmark system.

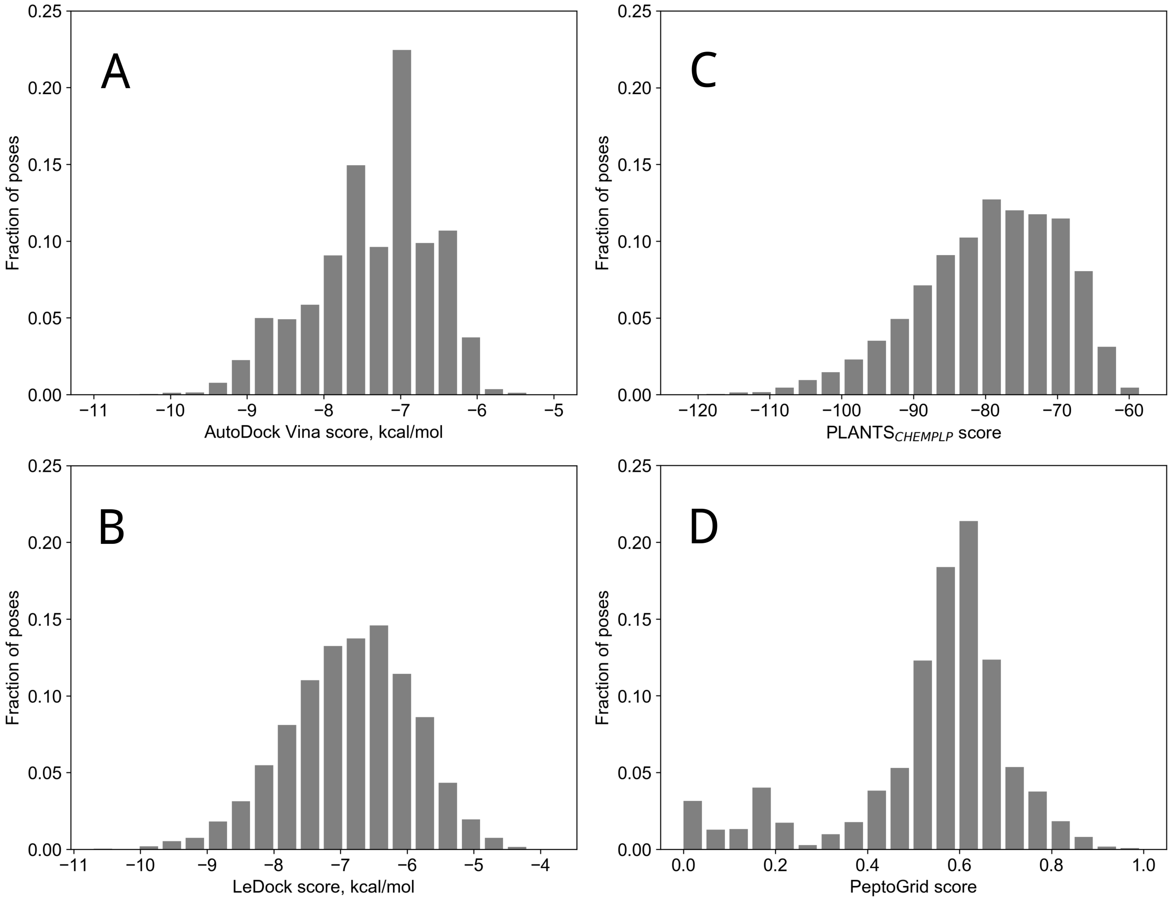

Figure S4. Distribution of docking scores. (**A**) AutoDock Vina energies, (**D**) LeDock energies, (**C**) PLANTS$_{\text{CHEMPLP}}$ score, (**B**) PeptoGrid score derived from AutoDock Vina.

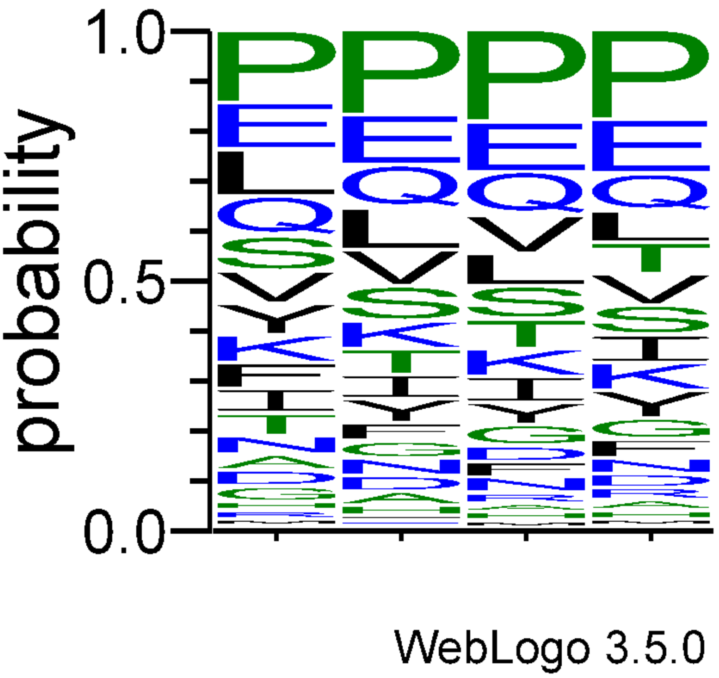

Figure S5. Logo of the tetrapeptide dataset used for GABAB screening system.

Table S1. Number of occurrences of peptides from top 20 poses.

Sequence	Number of occuerences	Maximum score
PSYG	6	0.97
PYYA	5	1.00
QFLG	2	0.99
RYPS	1	0.97
PVRG	1	0.96
SFSD	1	0.96
VFGK	1	0.95
FLGA	1	0.96
FFVA	1	0.96
PSFS	1	0.98

3. Apparatuses parameters

3.1. Open Field Test

The "open field" test was carried out in a 4L trapezoid tank. The parameters are shown in Figure 1. The base, back, and side walls are made of matte black plastic, and the front wall (of smaller length) is made of transparent acrylic glass.

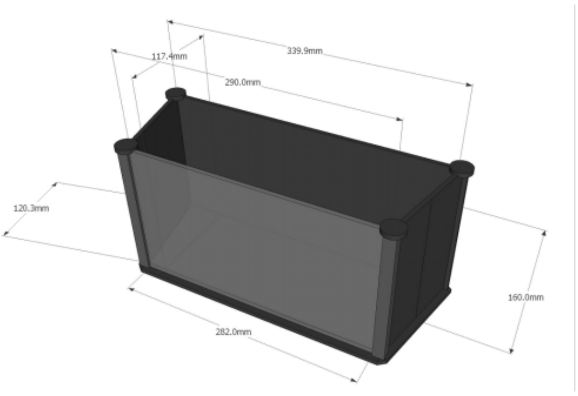

Figure S6. Scheme of the open field test tank.

The apparatus for shoal cohesion test is a plexiglass container with a removable partition (Figure S7). A "flock" of 5 adult *Danio rerio* individuals was placed in a small compartment, and the test fish was placed in a large compartment. The tank was illuminated by environmental light (about 200 Lx).

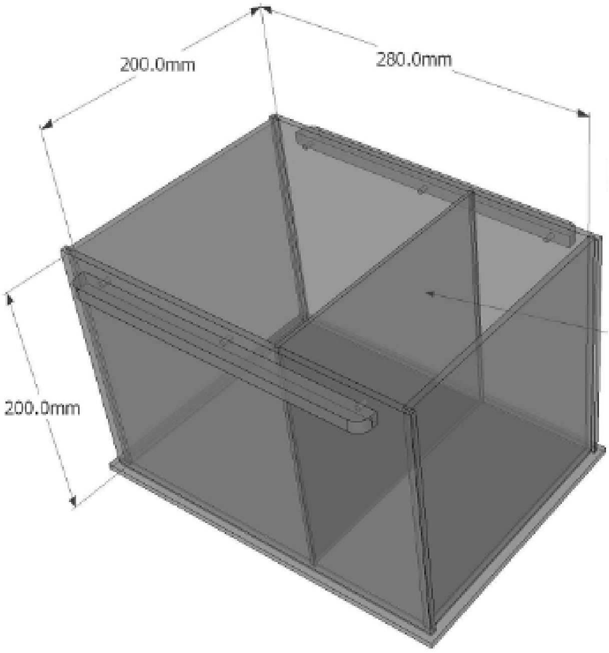

Figure S7. Scheme of the shoaling test tank.
